# Status and influencing factors of returning to work 6 months after discharge from hospital with severe acute pancreatitis-a cross-sectional descriptive-analytical study in China

**DOI:** 10.3389/fpsyt.2024.1280452

**Published:** 2024-05-17

**Authors:** Dengbi Yang, Mingtao Quan, Xuan Xiao

**Affiliations:** ^1^ Nursing Department, Affiliated Hospital of Zunyi Medical University, Zunyi, China; ^2^ Nursing College, Zunyi Medical University, Zunyi, China

**Keywords:** acute necrotizing pancreatitis, return to work, quality of life, intensive care units, anxiety, depression

## Abstract

**Objective:**

To describe the return to work of patients with severe acute pancreatitis within 6 months after discharge, and to explore the influence of demographic, clinical, and psychosocial factors on their return to work.

**Research design:**

Prospective 6 months follow-up study.

**Setting:**

A third class hospital in Guizhou Province. Adult of severe acute pancreatitis(18-60years), with a job before admission, in the intensive care unit ≥ 24 h, were included.

**Main outcome measures:**

To study return to work and influencing factors one, three and six months severe acute pancreatitis patients discharge. several measurements were used, including General Health Questionnaire (Demographic, disease-related, job-related and health behavior data), Readiness for Return-To-Work Scale and the Hospital Anxiety and Depression Scale.

**Results:**

Forty-three severe acute pancreatitis patients were included in our study, with mean age 41.53 years. Twenty-nine (67.44%) patients returned to work within 6 months, and fourteen patients did not return to work. The status of Readiness for Return-To-Work Scale: fourteen severe acute pancreatitis patients who did not return to work were mainly in the precontemplation dimension and prepared for action-self-evaluative dimension both 5 cases (35.71%), and the 29 patients who had returned to work were in the Proactive maintenance stage. The study showed that the independent risk factors for returning to work in SAP patients were chronic disease (OR, 0.095; 95% CI [0.011-0.822]; *p*=0.008), sepsis (OR, 0.071; 95% CI [0.015-0.339]; *p*=0.009), low education level (OR, 2.905; 95% CI [0.969-8.710]; *p*<0.001), and anxiety and depression at 6 months (OR, 1.418; 95% CI [0.996-2.019]; *p*=0.004).

**Conclusions:**

In conclusion, the return to work of patients with severe acute pancreatitis needs to be improved. Chronic diseases, sepsis, low level of education and higher degree of anxiety and depression at 6 months were important factors leading to their failure to return to work.

## Introduction

Intensive care unit patients experience a variety of treatment, care, and monitoring methods, such as tracheal intubation, hemodynamic monitoring, blood purification therapy, frequent blood tests, and immobilization, which increase the incidence of complications such as post-ICU syndrome and psychological disorders, resulting in an increased risk of death in ICU patients and a reduced quality of life after discharge (1–3). Returning to work is an important indicator of recovery after illness, and failure to return to work may increase the psychological burden of patients and bring economic pressure to families and society. K reviewed studies on the return to work of ICU patients from 2009 to 2018, and found that the rate of patients returning to work was 33.33% three months after discharge, and increased to 60% at 12 months. Moreover, patients who returned to work experienced unemployment, career change and deterioration of work conditions after returning to work. Factors affecting ICU patients’ return to work include gender, age, education, complications, mechanical ventilation, length of stay, anxiety, depression, social support, and type of work (4, 5).

Patients with severe acute pancreatitis have a sinister condition, many complications, easy to relapse, the case fatality rate is as high as 5% ~ 50%, and often need to be admitted to the intensive care unit for treatment (6–8). It is often accompanied by acute respiratory distress syndrome, renal function impairment, pancreatic encephalopathy, diabetes and other complications, thus extending the length of ICU stay, increasing the readmission rate and mortality (9). With the improvement of people’s living standards, the incidence of severe acute pancreatitis patients has increased year by year, and 50% of severe acute pancreatitis patients are of working age. A 2003 Finnish study of quality of life in patients with severe acute pancreatitis found that among 145 patients, patients under 63 years of age had a low rate of return to work (61%), but the follow-up time of patients was not uniform, and the study did not analyze the demographics of patients returning to work, disease and other relevant factors.

Therefore, this study followed up patients with severe acute pancreatitis after discharge from ICU to understand their status of return to work, and analyzed the impact of demographic factors, clinical and psychological factors on return to work.

## Materials and methods

### Study design and population

The study is a cross-sectional study, through 1, 3 and 6 months follow-up of ICU discharged patients with severe acute pancreatitis, to understand their return to work status and influencing factors. This study has been approved by the Ethics committee of the Affiliated Hospital of Zunyi Medical University(Ethics approval number:KLLY-2022-145).

The study subjects were SAP patients admitted to the ICU of a tertiary hospital in Guizhou Province from January 2022 to December 2022. Convenient sampling method was used to select the subjects. All participants were employed prior to enrollment, including full-time, part-time, agricultural, commercial, and private jobs.

### Inclusion criteria

(1)The main clinical diagnosis was SAP.
(2)Length of stay in ICU ≥48h.
(3)Age of study subjects: male 18-60 years old; Female 18-55 years old.
(4)Had a job before the illness.
(5)Informed consent and voluntary participation in this investigation.

### Exclusion criteria

(1) Patients who gave up treatment during hospitalization after diagnosis of SAP;
(2) Communication disorders, cognitive disorders or a history of mental disorders;
(3) have serious cardiovascular system dysfunction and physical defects cannot work;
(4) Patients who had retired or had no intention of returning to work at the time of inclusion.

### Sample capacity

According to the sample size calculation formula: N= Z2 * P* (1-P)/E2, Z=1.64, E=10%, P=0.5, N=67, which exceeded 5% of the original sample size, the sample size formula was adjusted to n ‘=n*N/(N+n-1). The minimum sample size included in this study was 27 cases.

### Research tool

(1) General Health Questionnaire

It was designed by the researchers according to the purpose of the study, including a total of 20 items of demographic data of the patients and disease-related data.

(2) The Readiness for Return-To-Work Scale (RRTW)

The Readiness for Return-To-Work Scale was developed by Dr. Franch of Canada (10), with 22 items, 13 items (1-13) for people who have not returned to work, and 9 items (14-22) for people who have already participated in work. It is divided into 6 dimensions, the people who have not returned to work have 4 dimensions: pre-intention, intention, action readiness - self-assessment, action readiness - action, and the people who have returned to work have 2 dimensions: uncertain maintenance, active maintenance, using Likert level 4 scoring. Cao Huali sinicized Readiness for Return-To-Work Scale (11). Cronbach’s α coefficient ranged from 0.75 to 0.84, and the retest reliability ranged from 0.80 to 0.883. The reliability and validity of the scale were good, and the dimension in which the patient scored higher was the dimension in which the patient was located.

(3) The Hospital Anxiety and Depression Scale (HADS)

The Hospital Anxiety and Depression Scale was developed by Zigmond and Snaith in 1983 to assess the current mental state of subjects and mainly screen inpatients for non-psychotic anxiety and depression symptoms (12). HADS includes two subscales of anxiety and depression. The Cronbach’s α coefficients of total scale, anxiety scale and depression scale were 0.879, 0.806 and 0.806, respectively, with a total of 14 items. Likert 4-level scores (0-3 points) were used for each item. When the index value of the Hospital Anxiety and Depression scale was 9 points, the sensitivity and specificity of the scale were the best, with >9 being positive and <8 being negative.

### Data collection

(1) The “General Health Questionnaire” was filled in by the researchers during hospitalization, and the online (wechat, telephone, etc.) follow-up, questionnaire (Qstar) was issued and collected by the researchers after discharge to investigate the status quo of SAP patients returning to work 1, 3 and 6 months after discharge;

(2) The Readiness for Return-To-Work Scale was filled in 6 months after discharge, 1-13 items were filled in for patients who had not returned to work, and 14-22 items were filled in for patients who had returned to work;

(3) The hospital anxiety and depression scale was filled in 1, 3, and 6 months after discharge.

### Data analysis

Microsoft Excel 2016 was used for data collection and sorting, and SPSS29.0 software was used for statistical analysis of the data. The measurement data were described by mean ± standard deviation, and the counting data were described by percentage. In the univariate data analysis, the measurement data (age, length of stay, anxiety and depression level) were used by two-independent sample t test, and the grade classification data (monthly family income, hospitalization expenses, education level) were used by non-parametric test. Chi-square test was used for binary classification (sex, smoking, drinking, first attack, mechanical ventilation, epidural anesthesia, acute respiratory distress syndrome, sepsis, and chronic diseases combined) and disordered multi-classification data (marital status, place of residence). Multiple linear regression analysis was used for multivariate analysis, with α=0.05 as the test level. Statistical value P<0.05 was considered statistically significant.

## Results

**Figure 1:**
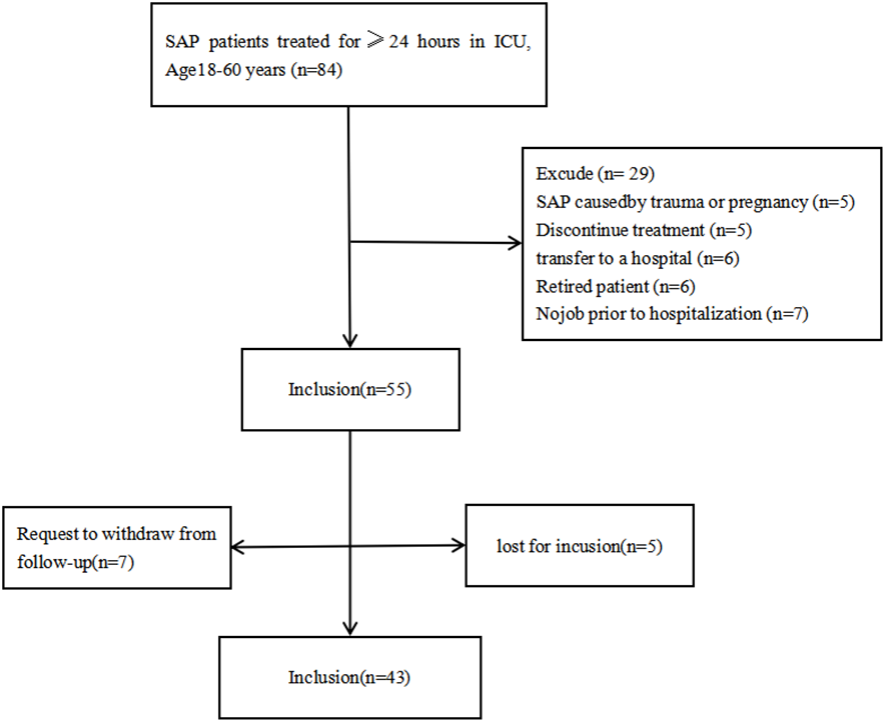
Flowchart of patients included in the study.

Forty-three SAP patients were selected according to inclusion and exclusion criteria(**Figure 1**), with mean age 41.53 (SD 10.93) years, 29 (67.44%) male. 33 (76.74) patients were diagnosed for SAP the first time, 25 (58.14) patients received mechanical ventilation and 19 (44.19%) patients received epidural anesthesia (The average duration is about six days) (**Table 1**).

**Table 1:** Demographic and clinical data for the study subject.

Characteristics	Mean (SD)	N (%)
Age (year)	41.53 (10.93)	
Sex		
• male		29 (67.44)
• female		14 (32.56)
Smoking		26 (60.47)
Drinking		23 (53.49)
Monthly household income		
• <3000yuan		8 (18.60)
• 3000-5000yuan		23 (53.49)
• 5000-10000yuan		10 (23.26)
• >10000yuan		2 (4.65)
hospitalization costs		
• Not affordable		11 (25.58)
• Can barely afford it		12 (27.91)
• Hardly a problem		17 (39.53)
• No problem at all		3 (6.98)
marital status		
• spinsterhood		3 (6.98)
• married		39 (90.70)
• divorced		1 (2.33)
Level of education		
• Primary and below		8 (18.60)
• middle school		22 (51.16)
• Technical secondary and high schools		5 (11.63)
• College or above		8 (18.60)
residence		
• Countryside		17 (39.53)
• towns		10 (23.26)
• downtown		16 (37.21)
Total hospital stay	17.44 (15.10)	
ICU hospital stay	11.91 (15.03)	
First-episode		33 (76.74)
mechanical ventilation		25 (58.14)
epidural anesthesia		19 (44.19)
Acute Respiratory Distress Syndrome		28 (65.12)
Sepsis		33 (76.74)
operation		7 (16.28)
Comorbid chronic disease		28 (65.12)

Statistics show that the rate of return to work was 30.23%, 55.81% and 67.44% and recurrence rates was 4.65%, 11.63% and 16.28% in 1, 3 and 6 months, respectively. 37.93% of SAP patients reported reduced work intensity after discharge, 91.3% of the patients had abstained from alcohol, and but 62.79% continued to smoke. The status of RRTW: The 14 SAP patients who did not return to work were mainly in the precontemplation dimension and prepared for action-self-evaluative dimension both 5 cases (35.71%)), and the 29 patients who had returned to work were in the Proactive maintenance stage (**Table 2**).

**Table 2:** RRTW status in patients with severe acute pancreatitis.

item		Score(Mean ± SD)
Had not returned to work(14)		
Precontemplation	5 (35.71%)	2.81 ± 1.21
Contemplation	3 (21.43%)	3.29 ± 1.00
Prepared for action—self-evaluative	5 (35.71%)	2.32 ± 0.60
Prepared for action—behavioral	1(7.14%)	3.14 ± 1.08
Has returned to work(29)		
Proactive maintenance	29 (100%)	3.52 ± 0.43
Uncertain maintenance	0 (0.00%)	2.32 ± 0.50

Univariate analysis results showed that compared to SAP patients who have returned to work, those who had not returned to work had higher monthly household income, lower levels of education, Less social support, higher levels of anxiety and depression at six months, combined chronic disease, and accompanied by sepsis (**Table 3**). Multiple linear regression showed that the independent variables affecting the return to work of SAP patients is combined chronic disease, accompanied by sepsis, levels of education and levels of anxiety and depression at six months (**Table 4**).

**Table 3:** Univariate analysis of demographic data of SAP patients whether to RTW at six-month follow-up.

Characteristics	Has returned to work (29)	Non-returned to work (14)	z/x2	P-value
Age (year)	40.93 ± 10.84	42.79 ± 11.40	-0.51	0.608
Sex			0.150	0.698
male	19 (65.52)	10 (71.43)		
female	10 (34.48)	4 (28.57)		
Smoking after discharge	12 (41.38)	4 (28.57)	0.663	0.512
drinking after discharge	2 (6.90)	1 (7.14)	0.573	0.449
Monthly household income			9.112	0.014
• <3000yuan	3 (10.34)	5 (35.71)		
• 3000-5000yuan	15 (51.72)	8 (57.14)		
• 5000-10000yuan	10 (34.48)	0 (0.00)		
• >10000yuan	1 (3.45)	1 (7.14)		
hospitalization costs				0.246
• Not affordable	5 (17.24)	6 (42.86)	3.229	0.208
• Can barely afford it	8 (27.59)	4 (28.57)		
• Hardly a problem	14 (48.28)	3 (21.43)		
• No problem at all	2 (6.90)	1 (7.14)		
residence			4.812	0.090
• Countryside	9 (31,03)	8 (57.14)		
• towns	6 (20.69)	4 (28.57)		
• downtown	14 (48.28)	2 (14.29)		
Level of education			8.850	0.021
• Primary and below	4 (13.79)	4 (28.57)		
• middle school	12 (41.38)	10 (71.43)		
• Technical secondary and high schools	5 (17.24)	0 (0.00)		
• College or above	8 (27.59)	0 (0.00)		
Total hospital stay	18.28 ± 17.67	42.79 ± 11.40	0.52	0.608
ICU hospital stay	12.21 ± 17.48	12.21 ± 17.48	0.19	0.853
First-episode	23 (79.31)	10 (71.43)	0.329	0.566
mechanical ventilation	15 (51.72)	10 (71.43)	1.506	0.220
epidural anesthesia	15 (51.72)	10 (71.43)	0.015	0.903
Acute Respiratory Distress Syndrome	19 (65.52)	9 (64.29)	0.006	0.937
Sepsis	6 (20.69)	8 (57.14)		0.035
operation	5 (17.24)	2 (14.29)	0.061	0.806
Comorbid chronic disease	16 (55.17)	13 (92.86)	6.107	0.013
January-HADS	12.79 ± 5.19	12.35 ± 6.39	0.24	0.812
March-HADS	5.68 ± 3.31	8.35 ± 6.40	-1.47	0.161
June-HADS	3.72 ± 2.43	7.86 ± 6.01	-2.48	0.026

**Table 4:** Multiple linear regression analysis predictors of patients returned to work at six months after SAP discharge.

	B	SE	β	t	P-value
Monthly household income	-0.105	0.080	-0.172	-1.324	0.194
Level of education	0.228	0.056	0.449	4.037	<0.0001
Comorbid chronic disease	-0.321	0.114	-0.321	-2.818	0.008
Sepsis	-0.264	0.096	-0.264	-2.750	0.009
June-HADS	-0.038	0.013	-0.349	-3.039	0.004

## Discussion

In this study, the return to work rates of SAP patients 1, 3 and 6 months after discharge were 30.23%, 55.81% and 67.44%, respectively, and some patients suffered reduced work intensity after returning to work. The return to work of SAP patients was mainly related to chronic disease, sepsis, anxiety and depression level 6 months after discharge and education level of patients.

In our study, the return to work rates is lower compared to Kimmo’s retrospective study (13), Kimmo’s study investigated SAP patients aged 16-74 years over a period of 1-8 years and primarily focused on the quality of life of SAP patients. In contrast, our study specifically tracked SAP patients aged 18-60 years at 6 months post-discharge from the ICU and dynamically examined their RTW status and health behavior at 1, 3, and 6 months. We also analyzed the influencing factors of returning to work. The study is valuable in enriching the clinical database for SAP patients.

In this study, patients with higher education level are more likely to return to work, and patients with vocational education or above all return to work. Consistent with the study results of Lee (14), the reason for the low return rate of patients with lower education level may be related to the status and role of patients in the unit and the job they are engaged in. People with relatively low education are often engaged in heavy physical work, which brings challenges and difficulties for patients with severe acute pancreatitis to return to work.

Patients with severe acute pancreatitis are often complicated with chronic diseases such as diabetes, hyperlipidemia and fatty liver, which affect their physical recovery. The results of this study showed that compared with patients with severe acute pancreatitis without chronic diseases, patients with chronic diseases were less likely to return to work. Kamdar’s investigation on ARDS patients returning to work after discharge from ICU also reported similar results (5). The rate of return to work 5 years after discharge for patients with no comorbidities at admission increased by 24% to 26% compared with those with comorbidities, while the rate of return to work with two comorbidities decreased by 16% to 18%. In this regard, it is of great significance to strengthen the treatment and management of comorbidities in patients with severe acute pancreatitis. Clinical staff should encourage patients to actively treat related diseases, strengthen follow-up of patients with severe acute pancreatitis with comorbidities, urge patients to review regularly, and improve their self-management ability.

The early onset of severe acute pancreatitis is rapid, rapid progression, systemic inflammatory storm can occur, and then multiple organ dysfunction; Systemic sepsis can be secondary to local infection in the middle and late stages, and is an important risk factor for death from severe acute pancreatitis (15). The results of this study showed that patients with sepsis during hospitalization had longer time to return to work after discharge or even could not return to work again, which suggests that medical staff should not only actively treat the complications of patients, but also pay attention to the rehabilitation of patients after discharge, and dynamically understand the social status of patients with severe acute pancreatitis after discharge from ICU.

The results of this study showed that the higher the anxiety and depression scores of patients with severe acute pancreatitis at 6 months after discharge, the lower the likelihood of returning to work. Studies have reported that the existence of depression and anxiety will have a negative impact on returning to work, especially in the case of physical illness (16), and patients who have experienced ICU rescue and treatment have a high incidence of post-ICU syndrome and post-traumatic stress disorder, which has a serious impact on the recovery of physical function and quality of life of patients (17). In addition, the particularity of severe acute pancreatitis disease is also an important reason for increasing patients’ anxiety and depression, research shows (18), 10% to 30% of patients with pancreatitis will have one or more recurrences after cure, and the harm of relapse is far greater than the first attack, 50% of patients need to be hospitalized again. In summary, anxiety and depression often affect the recovery of physical functions of patients, and even lead to depression, which seriously hinders the return of patients with severe acute pancreatitis to social life.

A number of studies on the return to work of ICU patients reported that male and young patients were more likely to return to work (19, 20), while the gender and age of patients with severe acute pancreatitis in this study did not have a significant impact on the return to work. The hospital may have included 67.44% of male patients in this study, while females accounted for a relatively small proportion. Compared with those who did not return to work, the age difference was small. Moreover, severe acute pancreatitis is an acute digestive tract disease, and some patients recovered their physical functions well after timely treatment.

### Study strengths and limitations

The strength of this study lies in its distinction from previous single-center retrospective cohort studies on ICU and SAP patients returning to work. Our study, however, is a single-center prospective cohort study, allowing for dynamic follow-up on the RTW of SAP patients. This approach enables a more timely and accurate evaluation of the RTW status of SAP patients who have been discharged from the ICU.

At present, few studies have been conducted on the return to work of SAP patients. The general data questionnaire in this study was developed according to the influencing factors of ICU patients’ return to work, which was not targeted, which may lead to incomplete inclusion factors. In addition, this study was a single-center study, which conducted a preliminary exploration on the return to work of SAP patients. In the future, the research area will be expanded and the sample size will be increased for in-depth research, so as to provide data support for promoting the return of SAP patients to society.

In addition, previous studies have showed that age, length of stay, and mechanical ventilation are predictors of return to work in ICU patients, but similar results were not obtained in this study. It may be due to differences in disease or a small sample size. In our study, 25.58% of patients were readmitted to hospital, and the reasons mainly included recurrence, gallstones and pancreatic cysts. Therefore, future studies can include these factors in the study of influencing factors.

## Conclusion

Our follow-up study indicated that the rate of RTW at 1, 3, 6 months was 30.23%, 25.58% and 18.60%, respectively. The influencing factors for SAP patients’ RTW at 6 months after discharge include the presence of education levels, combined chronic diseases, sepsis, as well as levels of anxiety and depression at the six-month mark. These findings suggest that clinical care should place greater emphasis on monitoring these factors. It is important to enhance clinical disease monitoring for SAP patients to minimize complications. Additionally, there should be strengthened health education programs for SAP patients with lower levels of education to reduce anxiety and depression. Encouraging active treatment of chronic diseases among SAP patients can also help mitigate the impact of these conditions on their ability to return to work.

## Data availability statement

The original contributions presented in the study are included in the article/supplementary material. Further inquiries can be directed to the corresponding author.

## Ethics statement

The studies involving human participants were reviewed and approved by Ethics Committee of Affiliated Hospital of Zunyi Medical University. The patients/participants provided their written informed consent to participate in this study. Written informed consent was obtained from the individual(s) for the publication of any potentially identifiable images or data included in this article.

## Author contributions

DY: Writing – original draft, Data curation, Investigation, Writing – review & editing. MQ: Supervision, Writing – review & editing. XX: Writing – review & editing, Project administration, Data curation, Writing – original draft.
